# A meta‐analysis of functional magnetic resonance imaging studies of divergent thinking using activation likelihood estimation

**DOI:** 10.1002/hbm.25170

**Published:** 2020-08-26

**Authors:** Lucy S. Cogdell‐Brooke, Paul T. Sowden, Inês R. Violante, Hannah E. Thompson

**Affiliations:** ^1^ School of Psychology University of Surrey Surrey UK; ^2^ Department of Psychology University of Winchester Winchester UK

**Keywords:** ALE, divergent, fMRI, meta‐analysis, semantic control network

## Abstract

There are conflicting findings regarding brain regions and networks underpinning creativity, with divergent thinking tasks commonly used to study this. A handful of meta‐analyses have attempted to synthesise findings on neural mechanisms of divergent thinking. With the rapid proliferation of research and recent developments in fMRI meta‐analysis approaches, it is timely to reassess the regions activated during divergent thinking creativity tasks. Of particular interest is examining the evidence regarding large‐scale brain networks proposed to be key in divergent thinking and extending this work to consider the role of the semantic control network. Studies utilising fMRI with healthy participants completing divergent thinking tasks were systematically identified, with 20 studies meeting the criteria. Activation Likelihood Estimation was then used to integrate the neuroimaging results across studies. This revealed four clusters: the left inferior parietal lobe; the left inferior frontal and precentral gyrus; the superior and medial frontal gyrus and the right cerebellum. These regions are key in the semantic network, important for flexible retrieval of stored knowledge, highlighting the role of this network in divergent thinking.

## Graphical abstract

A meta‐analysis of fMRI studies into divergent thinking, with a comparison to default mode, multiple demand and semantic control networks. This found four clusters; the left inferior parietal lobe; the left inferior frontal and precentral gyrus; the superior and medial frontal gyrus and the right cerebellum. The largest overlap is with the semantic control network, highlighting the role of this network in divergent thinking.

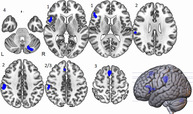

## 1 INTRODUCTION

Creativity is the result of a complex interaction between cognitive functioning, ability, personality, affect and motivation (Abraham, Rutter, Bantin, & Hermann, 2018). It is the foundation of our ability to progress; allowing us to interact appropriately with our ever‐changing environment. Thus, understanding the mechanisms that support creativity is of great interest. Definitions typically suggest that creativity requires the combination of *originality* (novel and unique; Runco & Jaeger, 2012) and *usefulness* (appropriate and meaningful; Runco & Jaeger, 2012), with some authors arguing for a third required element of *surprise* (Acar, Burnett, & Cabra, 2017; Boden, 2004; Simonton, 2012; Sternberg & Kaufman, 2018). Since Guilford's (1950) APA presidential address, ‘divergent thinking’ has been considered a key component of creativity (Onarheim & Friis‐Olivarius, 2013). It refers to the generation of many possible ideas for a particular problem. For example, in the Alternative Uses Task (AUT; Benedek, et al., 2014; Fink et al., 2010; Guilford, Christensen, Merrifield, & Wilson, 1960; Jung et al., 2010), one of the most commonly used divergent thinking task (DTTs), participants are instructed to generate as many alternative uses of conventional objects as they can (e.g., ‘name as many alternative uses for a BRICK as possible’). Tasks such as these, and other measures of creative thinking, have been widely used in conjunction with neuroimaging in recent years in an attempt to understand the neural bases of creative thinking. A great deal of initial work focused on the involvement of specific regions of the brain or on the neural time course of creative thinking (see reviews by Arden, Chavez, Grazioplene, & Jung, 2010; Dietrich & Kanso, 2010), however, it is also of interest to explore interactions of divergent thinking with large scale brain networks over time which few studies have sought to do (see Beaty, Benedek, Silvia, & Schacter, 2016).

Work on the brain regions involved in divergent thinking has tended to focus on three broad regions: the pre‐frontal, parietal and temporal cortices. These areas, as described below, are part of broader large‐scale brain networks which work together to shape cognition, and these networks are of particular interest in this meta‐analysis.

Several studies have implicated areas in the prefrontal cortex (PFC) as being important for divergent thinking, highlighting the requirement for executive and semantic control in this process, which rely heavily on prefrontal areas. However, there is some debate about the role of this region in divergent thinking. Some researchers suggest that the inferior frontal gyrus (IFG) is activated in divergent thinking, retrieving and selecting relevant remote associations for the production of *original* ideas, a process requiring *flexibility* (Abraham, Pieritz, et al., 2012; Abraham et al., 2018). Past meta‐analyses into divergent thinking appear to confirm this, with activation of the IFG being sensitive to semantic distance or associative strength (Wu et al., 2015). Other work reports that this activity seems weak. For instance, Fink et al. (2009, 2010) found that activation within the left IFG was only present when DTTs were compared to a fixation stimulus, but not when compared to control tasks. This may be because standard control tasks, such as object characterisation, also rely on the semantic control system, and so activity related to divergent thinking is not strong enough to survive the contrast. However, several studies have found an inhibitory role of the IFG (Beaty et al., 2016; Ivancovsky, Kleinmintz, Lee, Kurman, & Shamay‐Tsoory, 2018) with Kleinmintz et al. (2017) suggesting evaluating original ideas was related to an increase in activation in the left IFG, whereas generating original ideas was related to inhibition of the left IFG.The IFG has also been said to control activation in the middle temporal gyrus (MTG) said to be important in both the semantic and default mode network (DMN), with Vartanian et al. (2018) suggesting the IFG selects ideas that are generated by the MTG to produce responses consistent with the task demands.

More dorsal areas outside the realm of semantic control have also been associated with processes supporting divergent thinking. As part of a multiple demand network (MDN), areas such as the dorsolateral pre‐frontal cortex (DLPFC) allow the manipulation of information within working memory (Wagner, Maril, Bjork, & Schacter, 2001), as well as selecting and sustaining attention needed for fluency of responses (Shah et al., 2013). Some studies report increased activity in the DLPFC during creative tasks (Sun et al., 2016) with Abraham, Pieritz, et al. (2012) proposing the role of this region in creative processes. However, in other creative tasks such as improvisation, deactivation within executive control areas of the PFC corresponds to improvisational expertise (Limb & Braun, 2008; Pinho, de Manzano, Fransson, Eriksson, & Ullén, 2014), with deactivation of the DLPFC being said to contribute to increased cognitive flexibility (Nelson et al., 2007). Finally, previous studies have demonstrated anterior cingulate cortex (ACC) activated in many DTTs (Abraham, Pieritz, et al., 2012; Fink et al., 2009; Howard‐Jones, Blakemore, Samuel, Summers, & Claxton, 2005; Kleibeuker, Koolschijn, Jolles, De Dreu, & Crone, 2013) including the ventral anterior as well as posterior cingulate cortex (Mayseless, Eran, & Shamay‐Tsoory, 2015), and the both the anterior and posterior cingulate cortex has been shown to be key in the DMN (Beaty et al., 2014, 2020; Heinonen et al., 2016). The dorsal ACC particularly shows significant increase activity when creativity training is given (Sun et al., 2016), and an increase in activation has been noted across various divergent tasks (Abraham, Pieritz, et al., 2012; Kleibeuker et al., 2013) with this region implicated in the MDN (Duncan, 2010).This may reflect the role of ACC in conflict monitoring of prepotent but irrelevant responses (Botvinick, Cohen, & Carter, 2004). In summary, from the reviewed work both activation and deactivation in the PFC have been associated with divergent thinking and creativity.

There are also a number of studies that have focused on areas in the posterior parietal cortex in idea generation. Abraham, Pieritz, et al. (2012) and Fink et al. (2009) demonstrated that the left inferior parietal lobe (IPL), including the supra‐marginal gyrus (SMG), was important for the originality aspect of generating creative ideas using the AUT task amongst others. Benedek et al. (2018) showed that creating new ideas was associated with increased activation of the left SMG, supporting the role of the SMG in the left anterior IPL with generation of *original* ideas. The IPL has previously been associated with the use of the DMN in divergent thinking (Heinonen et al., 2016), and has been said to play a role, alongside other parts of the DMN, in producing new combinations important for originality during the creative process (Buckner, Andrews‐Hanna, & Schacter, 2008; Ellamil, Dobson, Beeman, & Christoff, 2012) with Ivancovsky et al. (2018) findings higher creativity in the generation phase of the AUT was associated with greater activation of the IPL, This area has also been previously associated with the verbal generation of ideas, and episodic memory retrieval during generation (Bechtereva et al., 2004; Mathias Benedek, Jauk, et al., 2014). The AUT task involves manipulation of common objects to find creative uses for them, and as previously mentioned the IPL is activated in these tasks. This region, however, has also been shown to be an area important for tool manipulation (Barde, Buxbaum, & Moll, 2007; Ishibashi, Ralph, Saito, & Pobric, 2011) and it is therefore, possible that the IPL is activated in response to mental manipulation of objects to aid the conception of novel or alternative uses. In contrast to the aforementioned work, other studies of divergent thinking, and related creative tasks, have shown deactivation within the right posterior parietal cortex, including the precuneus, superior parietal lobe (SPL) and right IPL (Gonen‐Yaacovi et al., 2013; Wu et al., 2015). It is currently unclear whether these results reflect the same network, or whether there were experimental reasons for the discrepancy of results.

The left posterior temporal cortex has also been implicated in divergent thinking. The fusiform gyrus (FG) as part of the visual network has been implicated in visuospatial creativity tasks (Chen et al., 2019) as well as construction of novel images and mental imagery (Chrysikou & Thompson‐Schill, 2011; Huang et al., 2013) with Yeh, Hsu, and Rega (2019) commenting that the left FG was activated during ‘incubation and insight’ and ‘evaluation and decision making’ along with the MTG. These regions have been shown to are both associated with object identification and naming (Martin & Chao, 2001), which may be important for *fluency* in tasks such as the AUT (Abraham, Pieritz, et al., 2012; Bechtereva et al., 2004; Fink et al., 2009, 2010). The middle temporal cortex (MTC) has been shown to be influenced by top‐down feedback from the PFC, which has strong links to DTT's and therefore this may link the middle temporal cortex to the generation of new ideas needed for *originality* (Wu et al., 2015). The posterior middle temporal cortex forms part of a semantic control network, alongside the PFC and dorsal angular gyrus (Noonan, Jefferies, Visser, & Lambon Ralph, 2013). Therefore, it may be important in bringing together remotely associated items, whilst inhibiting more dominant relationships.

What is clear from the above section is that a number of disparate brain regions respond to DTTs, and these areas are part of a broader network across the brain. There has been discussion in the literature as to the respective roles in divergent thinking and creativity of (a) bottom‐up thinking, allowing spontaneous and free‐flowing ideas, shown in the deactivation of key executive regions or response of areas classically defined as part of the ‘default mode’ network (Yeo et al., 2011) and (b) top‐down control to inhibit dominant responses and guide behaviour to be task appropriate, shown through activation of areas considered part of the MDN (Duncan, 2010), or executive control networks (Seeley et al., 2007) such as the dorsolateral frontal and parietal cortices.

The DMN is a distributed network of regions more active during rest, than during performance of attention demanding tasks, and is functionally defined by decreased activation during these tasks (Buckner et al., 2008). The four core regions identified in the DMN are the medial PFC, posterior cingulate cortex, and both the left and right IPL. Additionally the MTG is implicated as part of the DMN (Roger E Beaty et al., 2020; Buckner et al., 2008; Yeo et al., 2011). On the other hand, the MDN involves coordinated activity of a largescale network when taking part in goal‐directed effortful behaviour. This is known to activate regions such as the DLPFC, inferior frontal junction, and then dorsal and ACC (Crittenden, Mitchell, & Duncan, 2016; Duncan & Owen, 2000; Fedorenko, Duncan, & Kanwisher, 2013). Past research also points to the role of brain regions associated with cognitive control, known as the executive control network. This is engaged in tasks that require externally directed attention including the DLPFC, and anterior IPL (Beaty et al., 2016; Seeley et al., 2007), and these regions have been implicated in past meta‐analyses in divergent thinking (Gonen‐Yaacovi et al., 2013). The executive and DMN have been shown to cooperate in several processes involving top‐down modulation of information (Andrews‐Hanna, Smallwood, & Spreng, 2014).

These networks are well defined in literature, and there is agreement over network masks that are commonly used within neuroimaging research into these networks. (Yeo et al., 2011) produced seven cortical networks specified cortical parcellation using resting state functional, as well as a 17 network parcellation that split these networks into sub‐networks connectivity that identified the default mode and executive network. The MDN mask was taken from Duncan (2010) who produced this mask utilising previous reviews (Duncan & Owen, 2000) and these masks are regularly used throughout creativity literature (Beaty, Benedek, Kaufman, & Silvia, 2015; Evans, Krieger‐Redwood, Gonzalez Alam, Smallwood, & Jefferies, 2020; Lu et al., 2017; Mok, 2014) Recent work has begun to explore the interactions of these networks when performing DTTs and thinking creatively. In a review of this work, Beaty et al. (2016) propose that whereas the default mode and executive control networks normally act in opposition to each other, when thinking creatively a pattern of co‐operative activation emerges over time, with the salience network acting to co‐ordinate this coupling (see also Beaty et al., 2015). Given, the suggested importance of these networks for divergent thinking, the present meta‐analysis will compare overlap of the DMN and MDN, as well as the executive control network which has been included in an extended MDN (Camilleri et al., 2018), to areas activated in our meta‐analysis in order to make comparisons as to regions of similarity.

Of further interest is that between these networks sits a third, the semantic control network (Jefferies & Lambon Ralph, 2006) as defined in a mask (Noonan et al., 2013). This arguably is likely to play the greatest role in flexible thought (Jefferies, 2013). Semantic control may be important for divergent thinking because as a system it allows us to guide retrieval by inhibiting dominant associations and retrieving weaker relationships in a non‐automatic fashion (Lambon Ralph et al., 2017). Control is required when we: (a) retrieve weakly associated items, such as linking salt and sugar (Noonan, Jefferies, Corbett, & Lambon Ralph, 2010), (b) inhibit strong distractors, such as selecting piece goes with slice when cake is present (Noonan et al., 2010), (c) understand ambiguous words within the current context, such as bank at a riverside (Rodd et al., 2005), and (d) provide internally guided constraint when multiple potential responses are possible, such as during picture naming (e.g., Jefferies et al., 2008) or object use (Corbett, Jefferies, & Ralph, 2009). Regions implicated in semantic control have also been shown to be important in divergent thinking. The IFG within the ventral PFC is said to be critical for the selection of task‐relevant attributes (Stampacchia et al., 2018) as well as being important in the selection of distant associated in divergent thinking (Abraham et al., 2018). Vartanian et al. (2018) suggested that IFG selects these ideas that have been generated through activation in the MTG, which has also been shown to be needed for flexible processing of concepts in semantic activation (Hoffman, Pobric, Drakesmith, & Lambon Ralph, 2012; Whitney, Kirk, O'Sullivan, Lambon Ralph, & Jefferies, 2011). Interestingly, whilst the importance of semantic processing for divergent thinking has been a subject of continued interest (Beaty et al., 2020) and methods to analyse semantic relatedness of ideas produced in DTTs have been developed (Kenett, 2019), consideration of the role of the semantic control network has been largely separate from the neuroscience of creativity and divergent thinking literature. It has recently been argued that the semantic memory system may play an important role in creative thinking., largely due to the similarity of regions activated in these tasks and importance in the semantic control network (Gonen‐Yaacovi et al., 2013; Wu et al., 2015), however little research comments on the role of the semantic control network despite the overlap in regions known to be key in both this network and divergent thinking. Here we systematically synthesise and explore how much the neural mechanisms of semantic control overlap with those found to be involved in DTTs. We predict there will be extensive overlap between semantic control regions and areas which are found to be important for divergent thinking, such as left inferior frontal gyrus, posterior temporal and parietal regions. Although the semantic network, and the networks discuss above are functionally distinct, there may be overlap in regions that couple with other networks depending on the context.

A handful of meta‐analyses exist that have sought to summarise neural activity in creative tasks (Boccia, Piccardi, Palermo, Nori, & Palmiero, 2015; Gonen‐Yaacovi et al., 2013; Wu et al., 2015). Dietrich and Kanso (2010) firstly conducted a review of divergent thinking across EEG, ERP, and neuroimaging studies, finding highly variegated results, but changes in the ACC and prefrontal areas. Future studies sought to build upon this, with Boccia et al. (2015) examined domain specific creativity and found regions in the parietal frontal and temporal lobes were activated depending on the different domain: musical creativity activated MFG, left cingulate gyrus and IPL, whereas verbal creativity activated mainly the left hemisphere regions such as the PFC, middle and superior temporal gyrus and right IPL. Gonen‐Yaacovi et al. (2013) looked at creative tasks more generally and similarly found the lateral PFC, IPL and posterior temporal cortices were active. However, to our knowledge only one meta‐analysis specifically examining divergent thinking uses activation likelihood estimation technique (ALE; Wu et al., 2015). The ALE is a foci‐based technique, which treats foci as spatial probability distributions centred at coordinates rather than points, and seeks to estimate the likelihood of activations across multiple studies (Eickhoff, Bzdok, Laird, Kurth, & Fox, 2012; Laird et al., 2005; Turkeltaub, Eden, Jones, & Zeffiro, 2002). Wu et al. (2015) found regions important in divergent thinking could be split into the semantic and cognitive control systems, based on regions activated in their ALE, and therefore any replication should find similar results. Since the publication of Wu et al. (2015), the use of fMRI in creativity research has vastly increased with similar amounts published in the last 5 years compared to the 50 years before that. There has also been the creation of best practice guidelines for meta‐analyses, which aim to improve transparency, traceability, replicability and reporting (Müller et al., 2018). We therefore strongly believe an up to‐date meta‐analysis replicating Wu et al. (2015) but following the best practice guidelines is essential to provide a current consensus regarding the fMRI literature on divergent thinking. This will enable us to synthesise the disparate findings about the brain regions that are important, and more importantly discuss these regions within the context of existing networks, to explore an integration between work on the neural mechanisms of divergent thinking and those for semantic control

## 2 METHOD

### 2.1 Selection of studies

**FIGURE 1:**
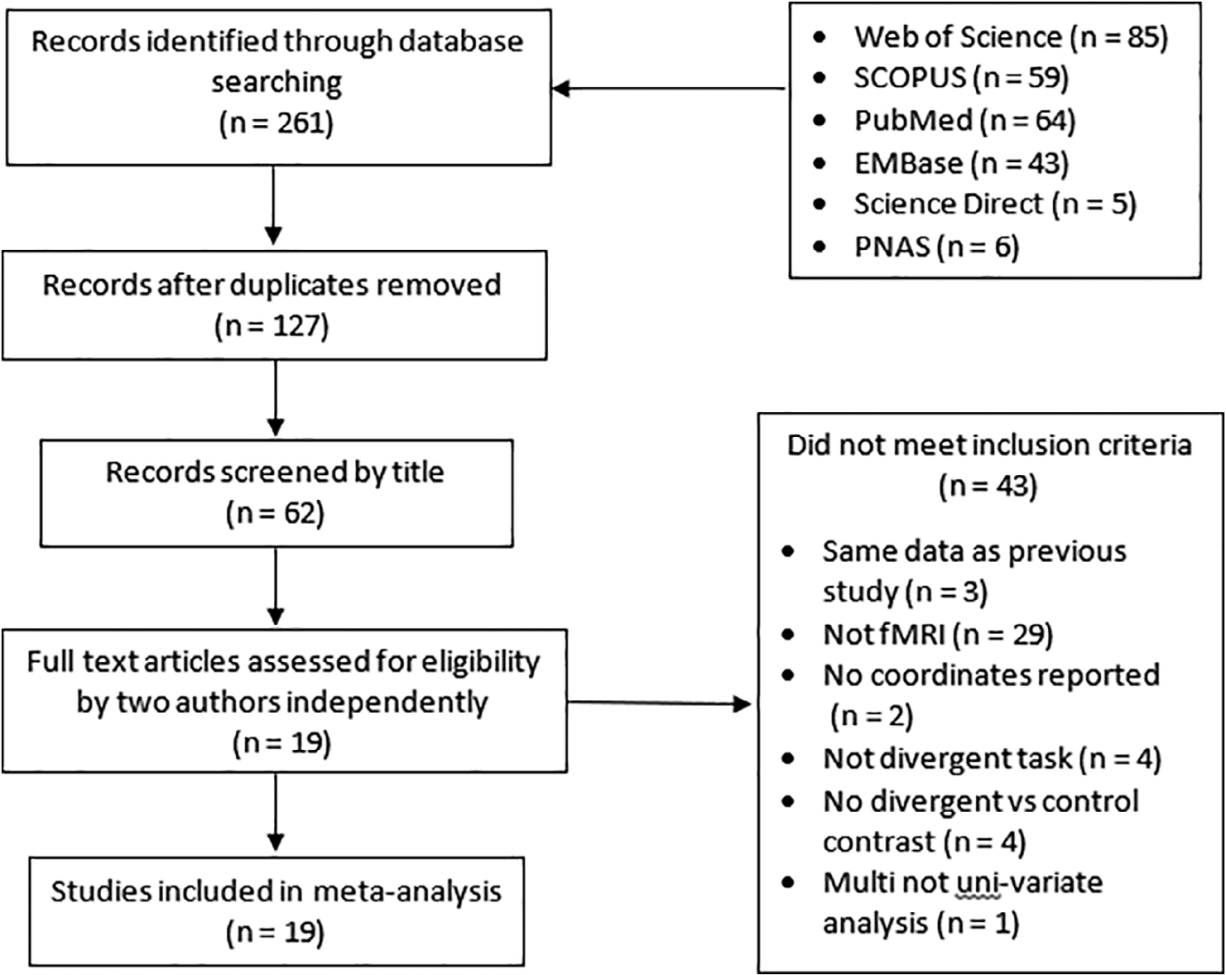
Modified PRISMA flow diagram showing the procedure followed for the meta‐analysis selection process. In all databases the title, abstract and keywords of the publication records were searched. All identified meta‐analyses reporting divergent thinking tasks using fMRI were screened. During assessment for eligibility all abstracts were checked for: (a) new data, (b) fMRI rather than other imaging methods, (c) results reported in full, (d) task was an established divergent task rather than novel experimental paradigm, (e) coordinates reported for contrasts needed

A systematic search was used to identify all literature in which healthy participants completed a DTT recorded by fMRI (Figure 1). An initial search was carried out on 19th March 2019 with the aid of PubMED, Scopus, PLOS, Web of Science, PsychINFO, ScienceDirect and EMBase databases using the following keywords in their title or abstract: creativ* AND (divergent AND thinking) AND (fMRI OR functional magnetic resonance imaging). This yielded 261 results across all databases, which were screened for inclusion in the meta‐analysis. These searches were re‐run in September 2019 to identify any new research meeting the criteria that had been published.

Inclusion criteria were as follows: (a) all subjects in the study were healthy adults, (b) all tasks tested divergent thinking in an experimental paradigm that also included control tasks, (c) all coordinates were reported in Montreal Neurological Institute (MNI) or Talairach space and (d) all reported activation coordinates were based on the entire brain.

After removing duplicates, 127 studies remained, which were reduced through screening by title to 62 studies. At this stage, further studies were searched for in reference lists of studies that passed screening, as well as previous meta‐analyses of fMRI studies of divergent thinking, however none were identified. Following this, two of the authors independently reviewed all study abstracts to select those that met the inclusion criteria. During this process, where contrast coordinates were identified as missing, authors were contacted a maximum of two times before a study was excluded. A total of 19 published fMRI studies passed all requirements and were taken forward to the ALE stage (Table 1), double the amount available in the previous meta‐analysis by Wu et al. (2015).

**TABLE 1:** fMRI studies for both divergent > control and control > divergent contrasts, and their regions of interest (CROI) selected in ALE meta‐analysis of divergent thinking *DTT > CT*

Author	Sample	Age: Mean	Conditions	ROIs	Foci	Response type, trial time	Evaluation of creative responses
Abraham, et al. (2018)	110	22.66	*Divergent condition* Div high AUT Div low: OL task *Control condition* Con high: 2‐back task Con low: 1‐back task	Div H > DivL (inclusive mask: DivH > ConH)	23	MR, 25 s per trial	Creative responses not evaluated but checked to verify acceptability of responses
Abraham, Piertiz, et al. (2012)	11	22.42	*Divergent condition* Div high: AUT Div low: OL task *Control condition* Con high: 2‐back task Con low: 1‐back task	Div H > DivL (inclusive mask: DivH > ConH)	15	MR, 25 s per trial	Creative responses not evaluated but checked to verify appropriateness of responses
Aziz‐Zadeh, Liew, and Dandekar (2013)	13	23.15	Creative: Creative visual task Control: Mental rotation task	Creative > control	10	SR	Creative responses not evaluated but categorised
Beaty et al. (2018)	29	21.79	Memory: recall past experience related to cue word Future: imagine novel event that could happen in the future Create: generate unusual use for object Sentence: construct a sentence based on two related words	Create > sentence	16	SR	Creative responses not evaluated
Beaty et al. (2017)	24	24.19	*Study phase* Cued recall task: recall noun‐verb pairings *Verb generation task* Low constraint: think creatively of a verb related to unseen noun High constraint: think creatively of a verb related to a cued recall noun *Control* Recall: recall verb when noun is shown from previous study phase	Low constraint > recall	4	SR	Created responses were coded for semantic distance via latent semantic analysis
Benedek, Beaty, et al. (2014)	28	26.20	Metaphor: produce a creative metaphor for an adjective Literal: produce a synonym for a metaphor	Metaphor > literal	8	MR, 10 s per trial	Metaphor responses evaluated for remoteness, novelty and cleverness by three raters on a 3‐point scale
Benedek et al. (2018)	42	24.31	*Create* Create original: AUT *Recall* Recall original: recall a nontypical original use they have previously encountered Recall common: recall a common use of object	Create original > recall common	6	SR	Creative responses judged by two raters on a 4‐point scale
Chrysikou and Thompson‐Schill (2011)	24	23.04	Uncommon use: generate novel use Common use: response with typical use Baseline: respond ‘yes’ if a black box is superimposed on an image, and ‘no’ if not	Uncommon use > baseline	3	SR	Creative responses evaluated for novelty and plausibility by two raters on 5‐point scale
Fink et al. (2015)	24	24.04	Creative: AUT Control: IT—instances task; generation of common and typical facts to stimuli	AUT > IT	4	MR, 15 s per trial	Creative responses evaluated for fluency and originality by four raters on 3‐point scale, top‐1 score used
Fink et al. (2009)	21	24.29	AU: completing AUT OC: completing object characteristics task NI: Name invention task; invent original names for fictional abbreviations WE: word ends task; complete word for given German suffixes	AU > OC	1	MR, 20 s per trial	Creative responses measured for fluency but not evaluated
Fink et al. (2010)	31	23.19	OC: completing object characteristics task AU: completing AUT AUinc: incubation condition; reflecting on responses given in AU condition AUstimu: cognitive stimulation condition; exposure to external ideas	AU > OC	1	MR, 21 s per trial	Creative responses evaluated for number and originality by four‐seven raters on 5‐point scale
Heinonen et al. (2016)	16	31.30	Idea generation: completing AUT Idea presentation: focus on AUT item with no production	Idea generation > presentation	3	MR, 15 min experiment	Creative responses evaluated for fluency, and reaction time measured
Ivancovsky et al. [2018]	36	27.53	GO: generation of original uses; AUT GC: generation of object characteristics	GO > GC	1	SR	Creative responses evaluated for fluency, flexibility and originality to produce mean score for each object
Japardi and Bookheimer (2018)	73	42.70	AU: completing AUT TQ: completing typical qualities; similar to GC	AU > TQ	9	MR, 20 s per trial	Creative responses scored for fluency and originality by six raters
Kleinmintz et al. (2018)	13	26.06	*Generation* GO: generation of original ideas; AUT GC: generation of object characteristics *Evaluation* EC: evaluation of object characteristics EO: evaluation of originality and appropriateness	GO > GC	4	SR	Creative responses not evaluated only reaction time measured
Mayseless et al. (2015)	26	25.70	AU: Completing AUT OC: Completing object characteristics task	AU > OC	2	SR	Creative responses evaluated for originality to produce average originality score per participant
Sun et al. (2016)	14	22.29	AUT: completing AUT OCT: completing object characteristics task	AUT > OCT	1	MR, 20 s per trial	Creative responses recorded after scanning and rated on 5‐point scale
Vartanian et al. (2013)	17	30.79	Generating uses: completing AUT ITI: inter trial interval (rest)	Generating uses > ITI	2	MR, 12 s per trial	Creative responses evaluated for fluency and reaction time
Vartanian et al. (2018)	44	35.47	Generating uses: completing AUT Recalling characteristics: Completing object characteristics task	Generating uses > recalling characteristics	13	MR, 12 s per trial	Creative responses evaluated for fluency during fMRI task, post scanning AUT repeated and scored for fluency, originality and flexibility
*CT > DTT*							
Author			Conditions	Regions of interest			Foci
Aziz‐Zadeh et al. (2013)			Creative: creative visual task Control: mental rotation task	Control > creative			5
Beaty et al. (2017)			*Study phase* Cued recall task: recall noun‐verb pairings *Verb generation task* Low constraint: think creatively of a verb related to unseen noun High constraint: Think creatively of a verb related to a cued recall noun *Control* Recall: Recall verb when noun is shown from previous study phase	Recall > low constraint			6
Fink et al. (2009)			AU: Completing AUT OC: Completing object characteristics task NI: Name invention task; invent original names for fictional abbreviations WE: word ends task; complete word for given German suffixes	OC > AU			1
Fink et al. (2010)			OC: completing object characteristics task AU: completing AUT AUinc: incubation condition; reflecting on responses given in AU condition AUstimu: cognitive stimulation condition; exposure to external ideas	OC > AU			2
Heinonen et al. (2016)			Idea generation: completing AUT Idea presentation: focus on AUT item with no production	Presentation > idea generation			9
Ivancovsky et al. (2018)			GO: generation of original uses; AUT GC: generation of object characteristics	GC > GO			3
Mayseless et al. (2015)			AU: completing AUT OC: completing object characteristics task	OC > AU			4
Sun et al. (2016)			AUT: completing AUT OCT: completing object characteristics task	OCT > AUT			5

Abbreviations: AUT/AU, alternative uses task; IT, instances task; OCT/OC, object characteristic task; OI, object identification; OL, object location task; Response type: MR, multiple responses; SR, single response; TQ, typical qualities.

### 2.2 Tasks

#### 2.2.1 Alternative uses task

The alternative uses task (AUT) task (Guilford et al., 1960), as previously described, is a widely used and well validated measure of divergent thinking (Benedek, et al., 2014; Fink et al., 2010; Jung et al., 2010; Kühn et al., 2014). Fluency, flexibility and originality are all measured. This task is commonly applied alongside control tasks, such as the object characteristics task, in which participants are instructed to generate features for the object presented, or object uses task where participants are instructed to name the use of the object presented (Kühn et al., 2014).

#### 2.2.2 Creative visualisation task

In this task, participants are asked to mentally manipulate three shapes to create a novel object (Aziz‐Zadeh et al., 2013). This task is often administered alongside a visuospatial control task such as the mental rotation task, where participants rotate an object to make a shape. Although involving visual processing, this task is divergent in nature as it requires novel responses, and is similar to AUT as it requires mental rotation of shapes, rather than an object, to produce something novel, with results being given verbally (Aziz‐Zadeh et al., 2013).

#### 2.2.3 Verb generation task

This task is similar to the AUT in that it requires a creative response to a stimulus, however here, stimuli presented are a series of nouns, and participants are asked to generate a novel verb related to the noun shown (Beaty, Christensen, Benedek, Silvia, & Schacter, 2017). Verb generation tasks have been shown to be a valid assessment of creative thought, as demonstrated by Prabhakaran, Green, and Gray (2014). They are often paired with a recall condition as the control task, where participants must recall previously studied noun‐verb pairs, which requires a convergent response.

#### 2.2.4 Novel metaphor task

In this task participants are required to create a metaphor that compares a topic to an unrelated object. (Benedek, et al., 2014) suggested that metaphor generation requires the formation of an abstract connection between two concepts, linking a conceptual category to a spontaneously generated other topic whilst ignoring relevant concepts. This open‐ended task therefore relies on similar cognitive processes to other divergent tasks. This task has been paired with a literal expression condition that matches the underlying processes but does not require divergent thinking (Beaty et al., 2017).

### 2.3 Activation likelihood estimation technique

ALE is a method utilised to integrate neuroimaging results from across studies (Laird et al., 2005; Turkeltaub et al., 2002) and has been used in previous meta‐analyses (Pidgeon et al., 2016; Wu et al., 2013). ALE models uncertainty in the localisation of activation foci, using Gaussian probability density distributions through modelling the probability distribution centred at the coordinates of each foci (Eickhoff et al., 2009). The size of the full‐width at half maximum (FWHM) of the Gaussian kernel is determined by an ALE algorithm, which accommodates larger samples sizes and therefore provides a more certain estimation of spatial locations. Probability distributions are combined into modelled activation maps, and activation probabilities, or ALE scores, which are calculated based on the union of maps across studies. ALE values are tested under the null distribution of spatial independence (Fitzgerald, Laird, Maller, & Daskalakis, 2008; Sabatinelli et al., 2011).

### 2.4 ALE analysis

This meta‐analysis was conducted using the revised approach from Eickhoff et al. (2012) following the latest recommendations for ALE meta‐analyses (Müller et al., 2018) using GingerALE 3.0.2. Software (http://brainmap.org/). Coordinates of the foci were taken from the original papers or from the experimenters directly providing the data. A total of 162 foci were reported in 19 experiments involving 596 participants.

In the ALE analysis of single datasets, regions of interest (ROIs) of fMRI studies on divergent tasks versus control tasks (127 foci reported in 19 experiments; Table 1) and control versus divergent tasks (35 foci reported in eight experiments; Table 1) were inserted separately. We also planned separate ALE analyses to assess the effect of different divergent tasks on the brain activity associated with divergent thinking, however only the AUT had enough studies to perform this and as there was no change in the overall pattern of results, the analysis will focus on all divergent tasks combined. Analyses were also were performed on studies requiring only one response (SR) versus multiple responses (MR) per trial (SR versus MR; 106 foci in 20 experiments) however this yielded no significant differences.

The ALE was run using MNI coordinates (peaks reported in Talairach space were converted to MNI using tal2icbm_spm transportation with GingerALE; Lancaster et al., 2007) according to the procedure proposed by Eickhoff et al. (2009). ALE maps were calculated using 5,000 permutations and a cluster level FWE of *p* < .05 with a cluster forming voxel level threshold of *p* < .001 based upon the latest recommendations from Müller et al. (2018). Only clusters with dimensions exceeding the recommended size were reported. Each ALE map was visualised using Mango (http://ric.uthscsa.edu/mango) and Anatomist (http://brainvisa.info/), and was overlaid on the anatomical MNI Colin27 template for visual inspection and representation purposes.

Following the initial analysis with a cluster forming voxel level threshold of *p* < .001, a follow‐up analysis was run using a cluster forming voxel level threshold of *p* < .01, which matches the threshold level of the previous meta‐analyses as well as the fMRI studies themselves.

### 2.5 Overlap analysis

We also compared the overlap of the ALE to pre‐existing brain networks, as we were particularly interested in whether these regions activated in divergent thinking overlapped with the networks, and to what extent. Network maps used were taken from existing and well established masks: default mode and executive control network from Yeo et al. (2011), MDN from Duncan (2010) and semantic control network from Noonan et al. (2013). Overlap was primarily a visual inspection of the resulting overlap maps. However, we also used the Dice Similarity Coefficient (DSC; Dice, 1945) a validation metric of spatial overlap between two segmentations. When there is no overlap DSC = 0 and complete overlap DSC = 1. Although we are not expecting to see a complete overlap, when DSC > 0 it shows evidence that regions activated in DTTs overlap with pre‐existing large‐scale networks.

### 2.6 Open access and declarations

The procedure followed for the meta‐analysis can be found in the Open Science Framework (OSF; https://osf.io/f5kxm/). Digital materials and data where possible are also available in the OSF (https://osf.io/h4qyu/) including full reporting of any analyses not reported. Any digit materials or data that are inaccessible due to the programme used can be released by contacting the corresponding author. The study was registered on OSF prior to beginning the systematic search and all manipulation and measure of this study are reported in the following sections.

## 3 RESULTS

Nineteen fMRI publications of divergent thinking, with an average sample size of 24.79 and a mean sample age of 28.59 were included in the present ALE analysis. Of these 190, eight included control > divergent contrasts.

### 3.1 ALE results of activated regions at *p* < .001

#### 3.1.1 DTT > CT

**FIGURE 2:**
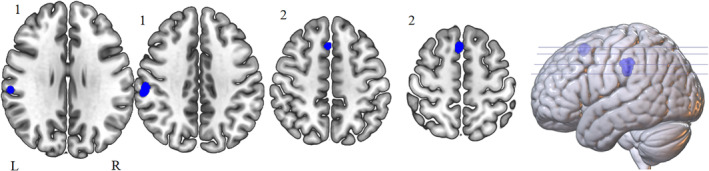
Peak ALE cluster locations for divergent thinking tasks > control tasks, activated at *p* < .001. Shown in neurological view, Cluster 1, labelled as such, is centred in the left parietal lobe (BA 40 and 6) and is 1,552 mm^3^ and Cluster 2 is centred in the left superior frontal gyrus (BA 6) and is 904 mm^3^

Table 2 and Figure 2 show the ALE results of fMRI studies of DTTs. Two clusters in the left hemisphere were more active under divergent tasks compared to control tasks. The peak ALE value of the first cluster was located in the left parietal lobe, with 50% in the post central gyrus, and 50% in the left IPL (BA 40; BA 2) [cluster coordinates are from (−64, −34, 28) to (−52, −18, 46) centred at (−58.8, −27.6, 37), with one peak with an ALE value of .021]. The second cluster peak ALE value was located in the left superior frontal gyrus (SFG; BA 6) [cluster coordinates are from (−6, 12, 48) to (2, 24, 60) centred at (−2.8, 18.5, 54.5) with one peak with an ALE value of .024]. No clusters that met the ALE threshold were located in the right hemisphere.

**TABLE 2:** ALE results for thresholding at *p* < .001

Cluster number	Peak	Region	Brodmanns area	*x*	*y*	*z*	ALE value	Contributors to cluster
*DTT > CT*								
1	1	Left inferior parietal lobe	40	−60	−26	32	.021	Abraham et al. (2018) Abraham, Pieritz, et al., 2012, Abraham, Beudt, et al., 2012 Beaty et al. (2018) Benedek et al. (2018) Fink et al. (2015) Fink et al. (2010) Vartanian et al. (2018)
	2	Postcentral gyrus	2	−58	−28	40	.020	
2	1	Left superior frontal gyrus	6	−2	20	54	.024	Abraham et al. (2018) Abraham, Pieritz, et al., 2012, Abraham, Beudt, et al., 2012 Aziz‐Zadeh et al. (2013) Beaty et al. (2017)
*CT > DTT*								
	No clusters found at *p* < .001							

#### 3.1.2 CT > DTTs

For control tasks versus divergent tasks, no clusters met the threshold level. This is likely due to there being only eight experiments that report activation foci for the contrast in this direction.

### 3.2 ALE results of activated regions at *p* < .01

#### 3.2.1 DTT > CT

**FIGURE 3:**
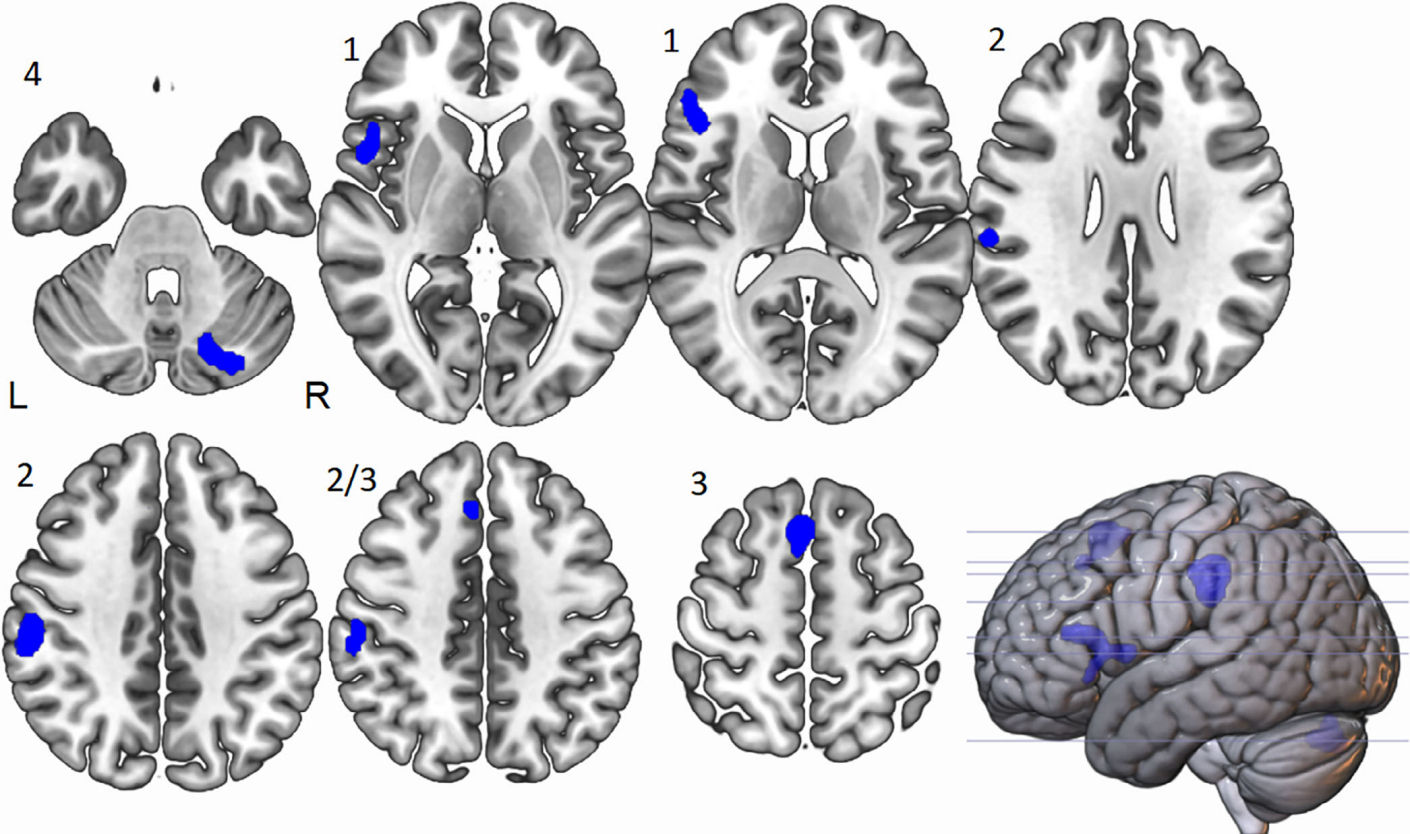
Peak ALE cluster locations for divergent thinking tasks > control tasks activated at *p* < .01. Shown in neurological view, Cluster 1, labelled as such, is centred in the left inferior frontal gyrus (BA 46) and is 1,904 mm^3^ in size, Cluster 2 is centred in the left inferior parietal lobe (BA 40) and is 2,288 mm^3^ in size and Cluster 3 is centred in the left superior frontal gyrus (BA 6) and is 1,944 mm^3^. Cluster 4 is centred in the right posterior cerebellum and is 1,600 mm^3^ in size

Table 3 and Figure 3 show the ALE results of fMRI studies in DTTs at the less conservative threshold level of *p* < .01. Four clusters were shown to be more active under divergent tasks than control tasks. The peak ALE value of the first cluster was located in the left IFG (BA 46) [cluster coordinates are from (−54, 6, −8) to (−42, 36, 18) centred at (−48.6, 21, 8.2) with five peaks with an overall ALE value of .012 which was not shown at *p* < .001. The ALE value of the second cluster was located in the left IPL (BA 40) [cluster coordinates are from (−64, −34, 26) to (−50, −18, 46) centred at (−57.6, −26.6, 36.8) with two peaks with an ALE value of .021, mirroring the cluster 1 at *p* < .001. The third cluster peak ALE value was located in the left superior frontal gyrus (BA 6) [cluster coordinates are from (−8, 8, 40) to (2, 30, 60) centred at (−3.2, 19.1, 53.3) with two peaks with an ALE value of .024 and again mirroring Cluster 2 from the ALE at *p* < .001. Finally, the fourth cluster with peak ALE value was located in the right hemisphere, in the right posterior cerebellum [cluster coordinates are from (16, −84, −36) to (6, −68, −22) centred at (24.7, −77.2, 30.7) with three peaks with an ALE value of .012] which was not shown at *p* < .001.

**TABLE 3:** ALE results for thresholding at *p* < .01

Cluster number	Peaks	Region	Brodmann area	*x*	*y*	*z*	ALE value	Contributors to cluster
*DDT > CT*								
1	1	Left pre‐central frontal gyrus	46	−50	10	6	0.12	Abraham et al. (2018) Abraham, Pieritz, et al., 2012, Abraham, Beudt, et al., 2012 Aziz‐Zadeh et al. (2013) Beaty et al. (2018) Kleinmintz et al. (2017) Vartanian et al. (2013)
	2	Left inferior frontal gyrus	46	−50	30	14	.012	
	3	Left inferior frontal gyrus	45	−46	22	10	.012	
	4	Left inferior frontal gyrus	47	−48	24	−2	.001	
	5	Left inferior frontal gyrus	47	−46	26	−6	.001	
2	1	Left inferior parietal lobe	40	−60	−26	32	.021	Abraham et al. (2018) Abraham, Pieritz, et al., 2012, Abraham, Beudt, et al., 2012 Beaty et al. (2018) Benedek et al. (2018) Fink et al. (2015) Fink et al. (2010) Vartanian et al. (2018)
	2	Left post central gyrus	2	−58	−28	40		
3	1	Left superior frontal gyrus	6	−2	20	54	.024	Abraham et al. (2018) Abraham, Pieritz, et al., 2012, Abraham, Beudt, et al., 2012 Aziz‐Zadeh et al. (2013) Beaty et al. (2017) Kleinmintz et al. (2017)
	2	Left medial frontal gyrus	8	−6	28	44	.011	
4	1	Right posterior cerebellum—Pyramis		28	−80	−32	.012	Abraham et al. (2018) Abraham, Pieritz, et al., 2012, Abraham, Beudt, et al., 2012 Beaty et al. (2018) Beaty et al. (2017) Benedek, Beaty, et al. (2014), Benedek, et al. (2014)
	2	Right posterior cerebellum—Uvula		26	−78	−26	.012	
	3	Right posterior cerebellum—Pyramis		20	−76	−32	.012	
*CT > DTT*								
No clusters found at *p* < .01								

#### 3.2.2 CT > DTT

Similarly to the results at *p* < .001, no clusters met the threshold level for the contrast control versus divergent tasks.

### 3.3 Cluster 1: The role of IFG in divergent thinking

When the divergent ALE mask was compared to a semantic control network (Noonan et al., 2013, Figure 4) we can see a number of areas of overlap, most notably with the IFG in Cluster 1. When comparing the ALE to the MDN (Duncan, 2010; Figure 5) the IFG also overlapped with this network. There was also overlap with both the executive control network and DMN (Yeo et al., 2011 Figures 6 and 7), emphasising the importance of both these networks as suggested in Beaty et al. (2016). This suggests that the DLPFC, which sits within the middle frontal gyrus in BA46, is a key area within cluster 1.

**FIGURE 4:**
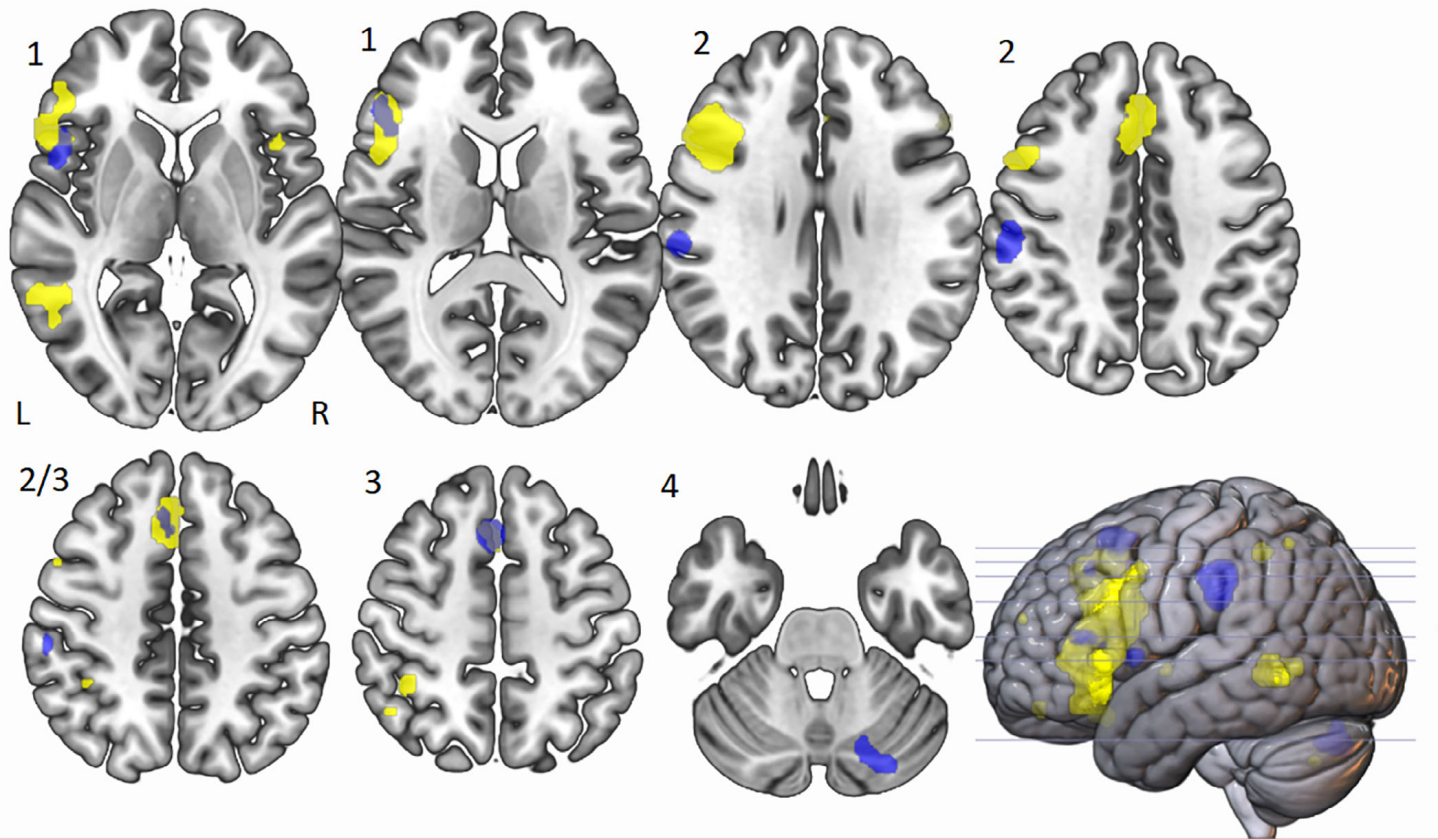
Neurological view of overlap of divergent ALE (blue) and semantic control system ALE from Noonan et al., 2013 (yellow) showing high Cluster 1 overlap (left IFG) and high Cluster 3 overlap (left SFG/MFG)

**FIGURE 5:**
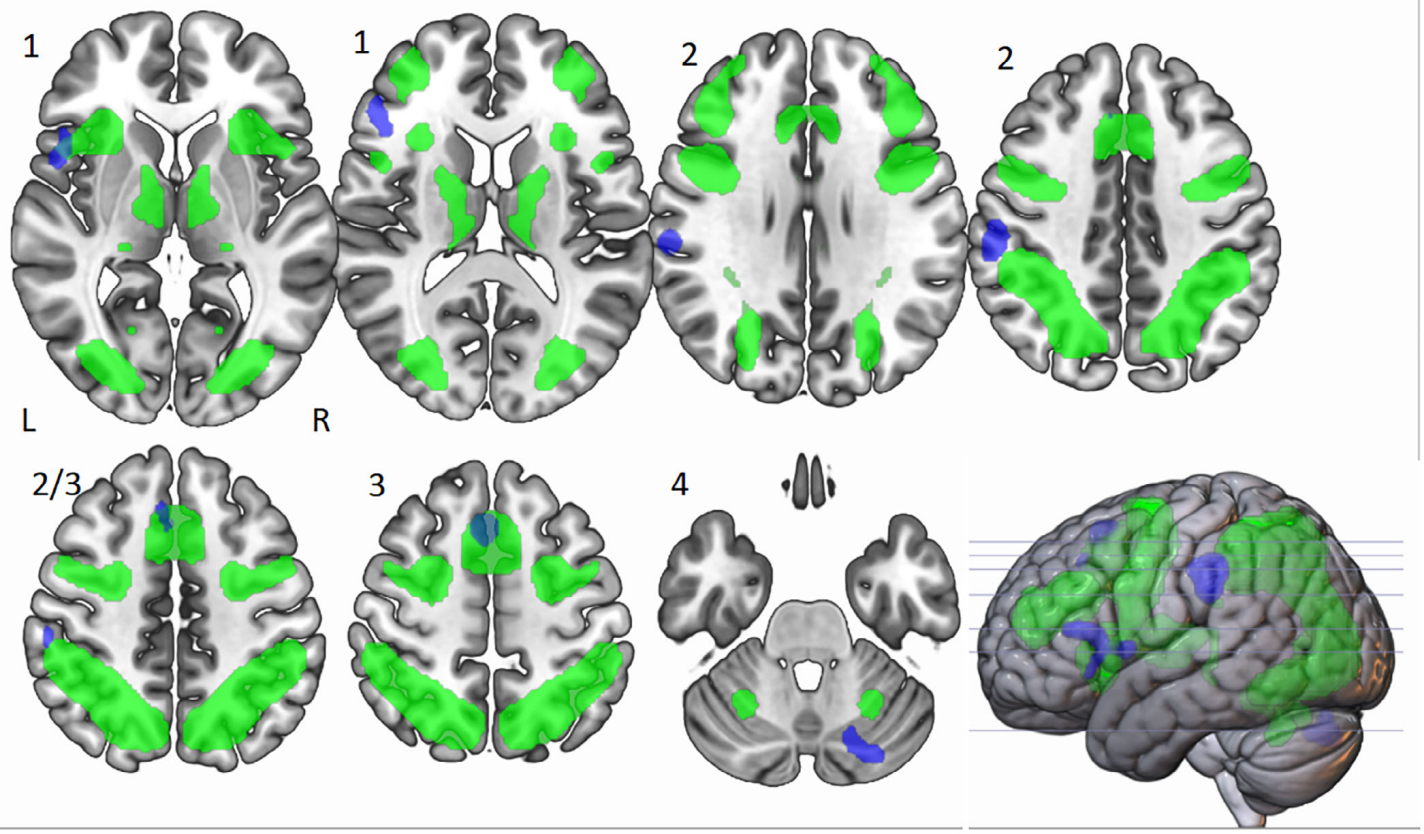
Neurological view of overlap of divergent ALE (blue) and multiple demand network from Duncan, 2010 (green) showing partial Cluster 1 overlap (left IFG) and high Cluster 3 overlap (left SFG/MFG)

**FIGURE 6:**
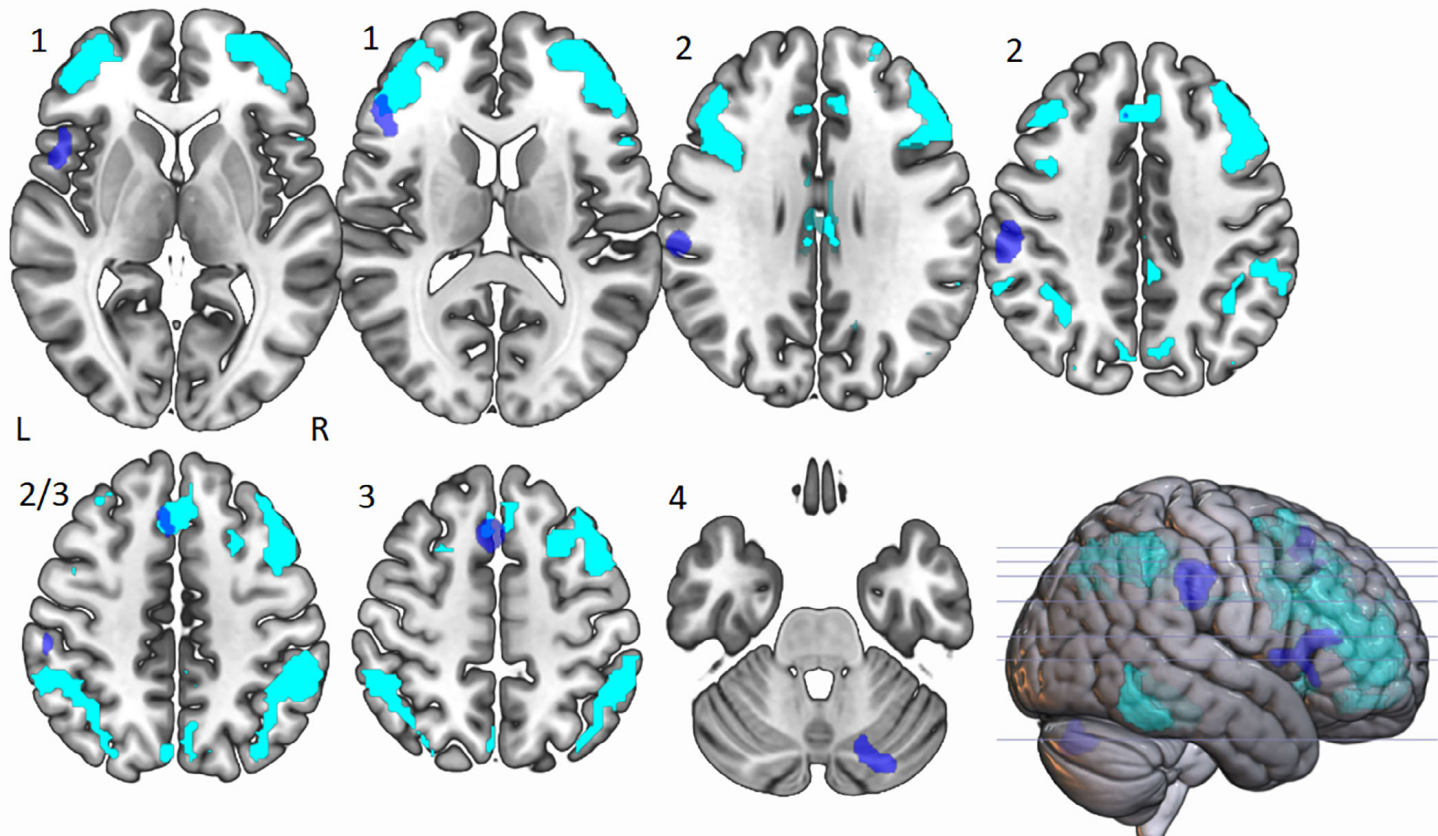
Neurological view of overlap of divergent ALE (blue) and executive control network from Yeo et al., 2011 (cyan) showing partial cluster 1 overlap (left IFG) and high cluster 3 overlap (left SFG/MFG)

**FIGURE 7:**
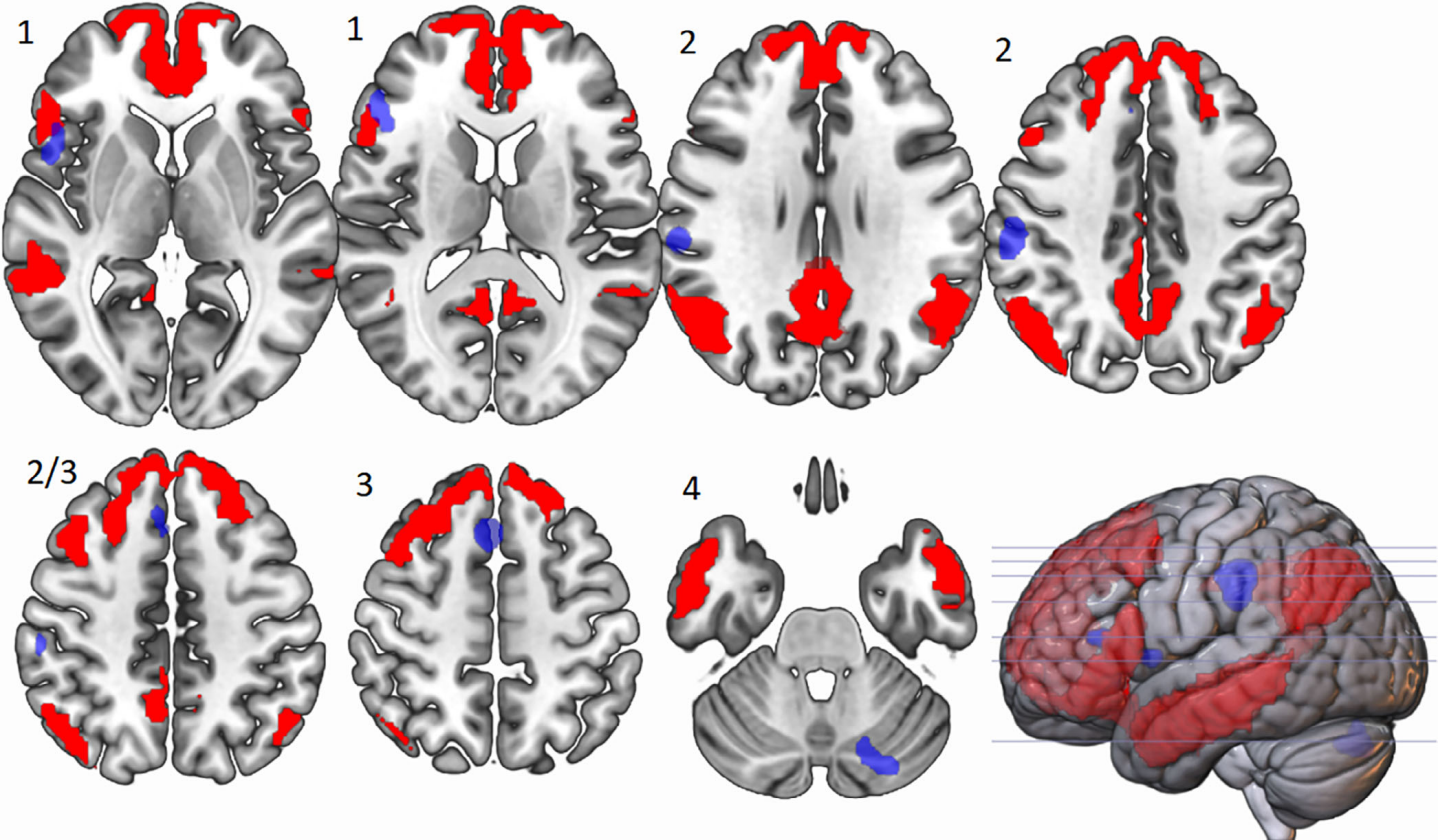
Neurological view of overlap of divergent ALE (blue) and default mode network from Yeo et al., 2011 (red) showing a small amount of Cluster 1 overlap (left IFG) and no overlap in Clusters 2 or 3

### 3.4 Cluster 2: The role of IPL in divergent thinking

**FIGURE 8:**
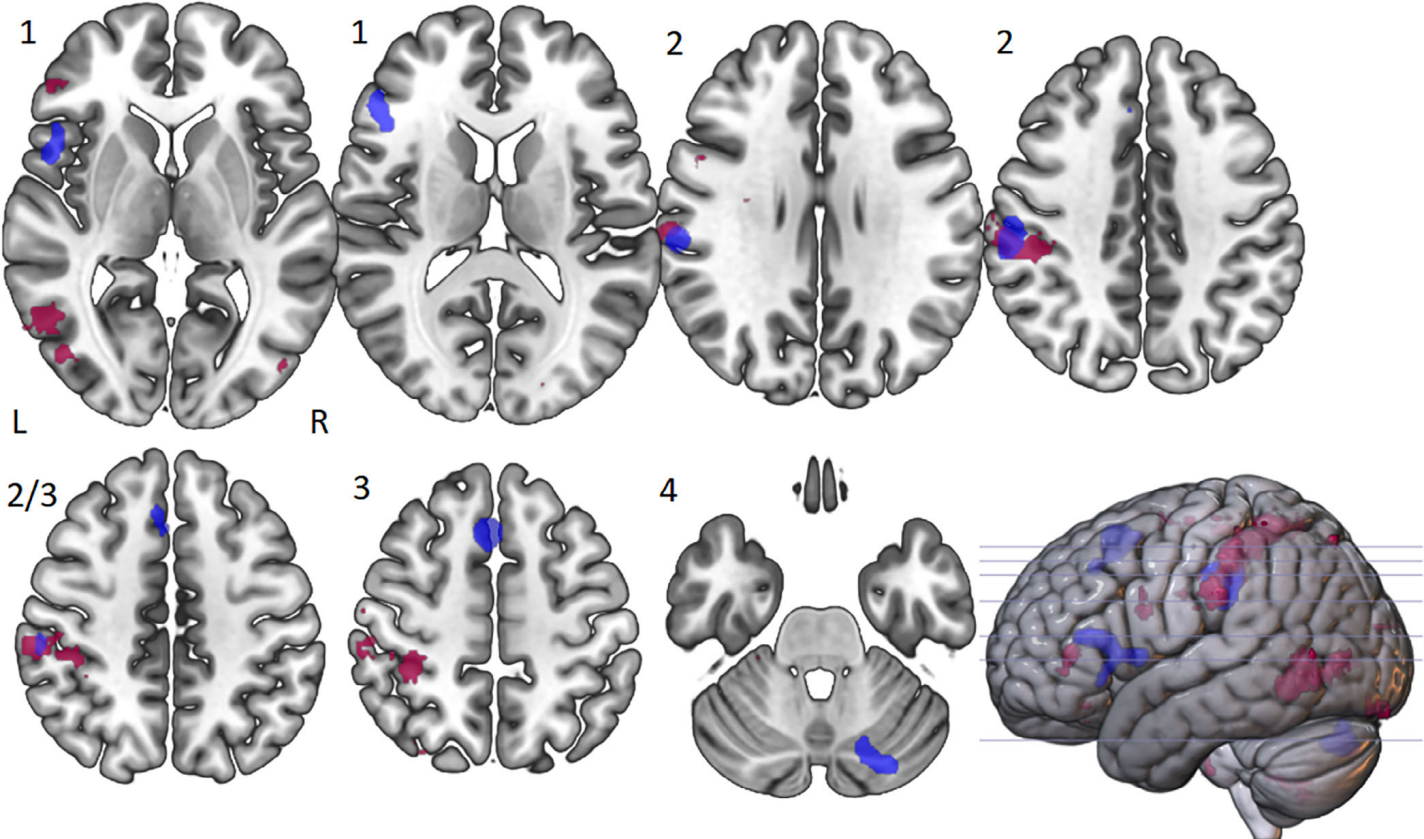
Neurological view of overlap of ALE (blue), and tools (pink) automated meta‐analysis of 115 studies produced using neurosyth (https://neurosynth.org/). Figures are labelled corresponding to clusters shown, with overlap in Cluster 1 (left IFG), Clutser 2 (left IPL) and Cluster 3 (left SFG/MFG)

We also compared Cluster 2 to the semantic control (Noonan et al., 2013; Figure 4), multiple demand (Duncan, 2010; Figure 5) and the executive and default mode (Yeo et al., 2011; Figures 6 and 7) networks. There was no overlap in any of the existing networks with the left IPL, and because of this we chose to compare this cluster to a mask for ‘tools’ as the IPL has been shown to be important in tool manipulation (Ishibashi et al., 2011), and the AUT involves processes similar to this. The ‘tool’ mask was created from the synthesis of 115 studies using Neurosynth software (https://neurosynth.org/; Figure 8), and we found a high level of overlap.

### 3.5 Cluster 3: The role of SFG/MFG in divergent thinking

When examining Cluster 3, we can see there appears to be overlap of the left superior and medial frontal gyrus with the semantic control system (Noonan et al., 2013; Figure 4).. We also observed overlap with the MDN, (Duncan, 2010; Figure 5), in which the left IFG and left MFG are closely linked. There was also an overlap with the executive control network (Figure 6) which may reflect activity in the DLPFC which sits in the middle frontal gyrus, and is key in BA 8 where Cluster 3 sits. There was no overlap with the DMN.

### 3.6 Cluster 4: The role of the cerebellum in divergent thinking

Only the semantic control system (Noonan et al., 2013) and MDN, (Duncan, 2010) showed any activation in the right cerebellum, however neither overlapped with cluster four.

#### 3.6.1 Dice similarity coefficient of ALE versus pre‐existing networks

When we compared the ALE clusters to pre‐existing networks, the largest overlap was with the semantic control network (DSC = 0.11). We also found similar overlap with the ALE and the tool meta‐analysis overlay (DSC = 0.09). The executive control and multiple demand both showed similar levels of overlap (DSC = 0.02) and the DMN showed the least overlap (DSC = 0.01).

## 4 DISCUSSION

In this study, an ALE meta‐analysis was conducted to explore the brain regions involved in divergent thinking. ALE results of 19 studies showed that the left IPL (BA 40) and left superior frontal gyrus (BA 6) were involved in creative idea generation in divergent thinking, with less stringent thresholding also implicating the left precentral, inferior and medial frontal gyrus (BA 44, 45/46 & 8). The putative roles of these regions for divergent thinking are discussed below.

### 4.1 Cluster 1—Left inferior and precentral frontal gyri (BA 45, 46, 47)

Our results indicate that the frontal cortex, more specifically the IFG were more active in DTTs. It is interesting that, despite often quite semantically demanding control tasks, these regions still appear to play a significantly greater role in divergent thought. Areas within the lateral PFC, such as the IFG, have been shown to be sensitive to the influence of the strength of the association between concepts (e.g., Noonan et al., 2010), allowing for the retrieval and selection of relevant associations to enable elaboration of concepts into novel ideas (Abraham, Pieritz, et al., 2012; Benedek, et al., 2014). The left IFG has a fundamental role in the semantic control network, which is widely replicated (cf, Noonan et al., 2013), and the overlap likely reflects the requirement to flexibly select weak and alternative associations between concepts whilst inhibiting the most dominant relationships needed for originality and fluency required for DTTs. This region also overlapped with the MDN; divergent tasks require constant attention towards the goal of the task, whilst supressing irrelevant or non‐creative ideas in order to increase fluency, and therefore the MDN may play a role in inhibition of this information and maintaining attention. These results suggest that the IFG assists in creative thought generation through retrieving loosely related semantic concepts and selecting creative ideas. Additionally, more dorsal prefrontal regions were found to be involved in divergent thinking. These regions, along with other more dorsal regions such as the DLPFC which sits within the middle frontal gyrus, were found to be significantly associated with divergent thought, and play a role in the MDN (Duncan, 2010) and the executive control network, where we also found overlap (Yeo et al., 2011). These areas are likely to play a role in completing the task by maintaining the task requirements through working memory, attention and inhibition (Abraham, Pieritz, et al., 2012; Fink et al., 2009; Kleibeuker et al., 2013).

### 4.2 Cluster 2—Left Inferior parietal lobe (BA 40)

The ALE results showed that the left IPL was more active under DTTs than control tasks, particularly in the AUT, which is consistent with the findings from (Wu et al., 2015). The left parietal region in BA 40 has been associated with actions and tool manipulation (Matheson, Buxbaum, & Thompson‐Schill, 2017; Matheson & Kenett, 2020). It is likely, therefore, that this reflects the role of the left IPL in the mental manipulation of objects displayed in DTTs such as the Alternative Uses paradigm, and has been cited as important in conceptual expansion (Abraham, Pieritz, et al., 2012). Connectivity between the IFG, which is required for controlled memory retrieval and response inhibition (Badre & Wagner, 2007; Beaty et al., 2014), and the IPL, was found to be higher in more creative individuals. As this cluster was found to be active in AUTs specifically, we can attribute the processes associated with the IPL to be particularly important in the AUT. Therefore, our analysis adds to the mounting evidence of the role of the left IPL within the generation of novel ideas, and we speculate that activation may be related to the buffering of relevant object knowledge needed during DTTs, especially the AUT.

### 4.3 Cluster 3—Left superior and medial frontal gyri (BA 6 and 8)

The superior and medial frontal gyri were also active in DTTs. This is likely to relate to the working memory demands involved in conceptualising and manipulating the relationship between an object and potential novel or unusual uses. Indeed, these areas of the frontal lobe have previously been associated with working memory (Abraham et al., 2018; Dietrich & Kanso, 2010; Du Boisgueheneuc et al., 2006). Whilst these regions have previously been found to be highly responsive to a range of semantic tasks (e.g., Binder, Desai, Graves, & Conant, 2009), they have also been found to be responsive more to high than low semantic control demands (Noonan et al., 2013), and are an integral part of the semantic control network. Nevertheless, their role extends beyond semantic tasks and appears to respond to difficult tasks which are non‐semantic (Duncan, 2010; Duncan & Owen, 2000; Vincent, Kahn, Snyder, Raichle, & Buckner, 2008). It has previously been speculated, therefore, that this region is important for ‘goal‐directed’ or top‐down retrieval (Binder et al., 2009). It may be that this region allows for the maintenance of the overall task goal. The SFG has been shown to play a role in working memory and could be activated in divergent tasks due to the need for flexibility in monitoring and manipulating semantic information into ideas that show originality. This region also overlaps with the pre‐sensory motor area, which suggests the role of motor stimulation in divergent thinking. Matheson and Kenett (2020) discuss that whilst the motor system executes actions, simulations of this system also support other higher‐order cognition such as creative tasks. Specifically, divergent tasks such as the AUT are served by simulations of actions, implemented in motor regions as well as being associated with tool use as previously discussed. The DLPFC, located within the middle frontal gyrus and shown to overlap with our third cluster has been shown to be important in the semantic control network, with a role in integrating semantically distant information, and creative idea selection, needed to search in depth for higher‐level connections (Lucchiari, Sala, & Vanutelli, 2018) as well as the executive control network where it is proposed to exert top‐down influence over generative processes (Beaty et al., 2016). The MFG is a key region in the MDN (Duncan, 2010), and is likely to play a role, alongside the IFG, in the suppression and inhibition of ideas that are not suitable in order to remain task focused on those that have originality, providing further fluency of responses (Abraham, Beudt, et al., 2012; Fink et al., 2009; Kleibeuker et al., 2013).

### 4.4 Cluster 4—Right cerebellum

This meta‐analysis found activation in the right hemisphere only in the parietal cerebellum. The cerebellum was found to be activated in numerous studies (Abraham et al., 2018; Anna Abraham, Pieritz, et al., 2012; Beaty et al., 2018; Benedek, et al., 2014), however, very little discussion is given as to why this may be. The cerebellum itself has historically been implicated in motor control, however, the conception of the cerebellum has progressively evolved to that of a modulator of cognitive functions to which it is reciprocally connected (Andreasen & Pierson, 2008; Marien, Engelborghs, Fabbro, & De Deyn, 2001; Stoodley & Schmahmann, 2010). However, it has also been said to play a role in the phonological loop function, a store that can hold verbal memory traces and a re‐articulation rehearsal process that refreshes memory traces (Baddeley, 2003). Takeuchi et al. (2017) suggested that the language related functions of the cerebellum are important for the effective production of ideas in verbal divergent thinking, and that reciprocal connectivity to language‐related areas, with the posterior lobe particularly being implicated in higher order processes such as phonological, semantic and word generation (Stoodley & Schmahmann, 2010). As many of the divergent tasks involved verbal responses, this may explain activation of this area.

### 4.5 Whole brain networks

Our comparison to clearly defined whole brain networks found several points of key interest. Firstly, there was the largest overlap according to DSC with the semantic control system (Noonan et al., 2013), from Clusters 1 and 3. This highlights the importance of a flexible semantic retrieval to the process of divergent thinking, requiring dampening down prepotent responses in order to actively select non‐dominant but task relevant information. Secondly, there was partial overlap of the multiple demand and executive networks (Duncan, 2010; Yeo et al., 2011), and a small overlap with the DMN (Yeo et al., 2011). This is in line with Beaty et al.'s finding of coupling between these networks during creative processing (Beaty et al., 2016). This may reflect the importance of the default mode system in producing new combinations of concepts, important for originality (Buckner et al., 2008; Ellamil et al., 2012), while the executive system maintains top‐down constraint to maintain the overall task goal.

### 4.6 Comparison to previous findings

As our meta‐analysis was more stringent than Wu et al. (2015), we included just four of the 10 studies in their analysis, with the remaining six being screened out. The first broke our inclusion criteria regarding the need for adults in the sample (Kleibeuker et al., 2013), two further studies focused on improving or evaluating creativity which were not the focus of this meta‐analysis (Fink et al., 2012; Mashal, Faust, Hendler, & Jung‐Beeman, 2007), one did not use a control task rather comparing new versus old ideas (Benedek, et al., 2014) and the final two involved creative story generation, which was not included due to the complex other processes require to write creatively, adding too much noise to the analysis (Howard‐Jones et al., 2005; Shah et al., 2013).

There were some discrepancies between our results and Wu et al. (2015). This ALE found additional clusters within both the superior and medial frontal gyrus (BA 6 & 8) which were not present in Wu et al. It could be that in the present meta‐analysis the majority of tasks had a heavy semantic basis, and there was less variation in the tasks that met the inclusion criteria, increasing the power. These regions could also be activated in this present research due to the addition of 12 studies, which may have led to enough foci to allow these regions to show as activated, compared to only 10 in the previous study. We also did not observe areas of activation at either threshold levels within the semantic system in either the left MTG (BA 39) or the left FG (BA 37), areas said to be important in the activation of long term memories related to idea generation, nor did we observe activation in the right ACC (BA 32) which has been associated with the suppression of irrelevant thoughts (Anderson et al., 2004; Howard‐Jones et al., 2005). This could be due to the exclusion of creative writing tasks, and these regions could particularly be important in these due to the use of long‐term memories in story generation, and the ongoing need to supress irrelevant thoughts during a task that requires focus for an extended period of time. Wu et al. (2015) also described deactivation of regions in the right posterior parietal regions (IPL & SPL; BA 40 & 7) and the right MFG (BA 46), which they explained as focusing attention to the most important processes in creative idea generation. This deactivation was then proposed to interact with posterior parietal regions involved in inhibiting irrelevant processes. However, the current meta‐analysis found no evidence to support this, and we were not able to run any analyses on controls versus divergent thinking due to a lack of foci required to find activation in any areas. This could be due to the lack of studies that report contrasts in this direction, or the addition of recent studies, which use a variety of control tasks, leading to a spread of activations across inconsistent regions.

Analyses were also were performed on studies requiring only one response versus multiple responses per trial, which yielded no significant differences. This suggests that there are no differences, that we could detect, in brain regions activated during single compared to multiple responses. This supports developments in divergent thinking research; more efficient fMRI tasks can be run that focus on single responses, leaving more time to conduct more trials, or test multiple concepts without needing longer multiple response trials.

### 4.7 Limitations and future directions

This ALE analysis of fMRI studies of divergent thinking, is the first in this field to follow strict guidelines on conducting fMRI meta‐analyses. Nonetheless, it was limited in scope because a number of the studies included were conducted before the creation of best practice procedures (Eickhoff et al., 2012; Müller et al., 2018). In order to identify key ROIs, running an additional less conservative analysis assisted in a more accurate picture of where the literature currently sits. Divergent thinking, an aspect of creativity, which is one of the most difficult psychological phenomena to quantify scientifically, was tested using a range of methods. Notably, nine of the 20 studies did not require the subject to produce an overt creative response, but to think through creative response options, which were not recorded. Whilst recording responses in fMRI is challenging, one of the difficulties of not recording responses is that you cannot evaluate the creativity of the response. Additionally, for studies that did record the creative responses, regions active during the task were included in the analysis irrespective of the response. We know that creativity involves multiple components, and the long‐time blocks included in each analysis reflect this. Previous studies have revealed that particular regions are more responsive to certain aspects of creativity than others. One aspect of creativity is the evaluation of ideas, as well as the idea generation themselves. Evaluations of creativity have been said to entail several processes different to idea generation (Coubard, Duretz, Lefebvre, Lapalus, & Ferrufino, 2011). For example, the left IFG has been found to be more activated during evaluations of ideas than during generation (Kleinmintz et al., 2017) and therefore peaks reported in this ALE that include the left IFG could reflect one or several creativity components. It is not possible to distinguish these roles within the current meta‐analysis.

When comparing divergent tasks to a control, we are aiming to isolate the regions that are activated during the specific task, reflecting the processes that are taking place. However, a limitation of using control tasks, especially with DTTs, is all the control tasks rely on the semantic system. For example, the control task commonly associated with the AUT is the object characterisation task and required the semantic system to be able to recall and name characteristics of objects presented. We therefore may be cancelling out any activity that is relevant for assessing the relationship between divergent thinking and semantic control because of this. A study directly comparing the two concepts would be critical to elucidate the similarities and differences of these networks.

Finally, an important distinction should be made between activity and connectivity of regions. This current meta‐analysis is able to comment on activity in regions during divergent tasks, however it is unclear whether this is as a direct result of the task itself, or whether functional coupling with other regions is the cause. More recent research has shown that functional coupling of the DMN and executive network supports creative idea production, particularly in older adults (Adnan, Beaty, Silvia, Spreng, & Turner, 2019; Beaty et al., 2015) and therefore it is possible some regions we reported activity in are activated due to their connectivity with other regions, rather than as a result of the task itself. A dynamic causal modelling approach would be needed to suggest directional effective connectivity, and future research focusing on which regions show activity, and which connectivity, would provide important further insight into the neural correlates of divergent thinking.

## 5 CONCLUSION

This meta‐analysis is the first to explore activity to DTTs in the broader context of the semantic control network, as well as relating these regions to the default mode, executive control and MDNs. This analysis revealed a significant relationship between activity to divergent thinking and the semantic control network. There was an additional role for the online mental manipulation of objects from the IPL. Therefore, a distributed network is implicated in divergent thinking. However, it is likely that the regions are at least partially specialised, given the partial but not complete overlap with the semantic control network. This possibility remains to be further explored.

## CONFLICT OF INTEREST

The authors confirm there are no potential sources of conflict of interest.

## DATA AVAILABILITY STATEMENT

The procedure followed for the meta‐analysis can be found in the Open Science Framework (OSF; https://osf.io/f5kxm/). Digital materials and data where possible are also available in the OSF (https://osf.io/h4qyu/) including full reporting of any analyses not reported. Any digit materials or data that are inaccessible due to the programme used can be released by contacting the corresponding author. The study was registered on OSF prior to beginning the systematic search and all manipulation and measure of this study are reported in the following sections.
